# Word Naming in the L1 and L2: A Dynamic Perspective on Automatization and the Degree of Semantic Involvement in Naming

**DOI:** 10.3389/fpsyg.2017.02256

**Published:** 2018-01-17

**Authors:** Rika Plat, Wander Lowie, Kees de Bot

**Affiliations:** ^1^Department of Applied Linguistics, University of Groningen, Groningen, Netherlands; ^2^Unit for Language Facilitation and Empowerment, University of the Free State, Bloemfontein, South Africa; ^3^Department of Applied Linguistics, University of Pannonia, Veszprém, Hungary

**Keywords:** semantic processing, word naming, L2, language processing, automatization and control

## Abstract

Reaction time data have long been collected in order to gain insight into the underlying mechanisms involved in language processing. Means analyses often attempt to break down what factors relate to what portion of the total reaction time. From a dynamic systems theory perspective or an interaction dominant view of language processing, it is impossible to isolate discrete factors contributing to language processing, since these continually and interactively play a role. Non-linear analyses offer the tools to investigate the underlying process of language use in time, without having to isolate discrete factors. Patterns of variability in reaction time data may disclose the relative contribution of automatic (grapheme-to-phoneme conversion) processing and attention-demanding (semantic) processing. The presence of a fractal structure in the variability of a reaction time series indicates automaticity in the mental structures contributing to a task. A decorrelated pattern of variability will indicate a higher degree of attention-demanding processing. A focus on variability patterns allows us to examine the relative contribution of automatic and attention-demanding processing when a speaker is using the mother tongue (L1) or a second language (L2). A word naming task conducted in the L1 (Dutch) and L2 (English) shows L1 word processing to rely more on automatic spelling-to-sound conversion than L2 word processing. A word naming task with a semantic categorization subtask showed more reliance on attention-demanding semantic processing when using the L2. A comparison to L1 English data shows this was not only due to the amount of language use or language dominance, but also to the difference in orthographic depth between Dutch and English. An important implication of this finding is that when the same task is used to test and compare different languages, one cannot straightforwardly assume the same cognitive sub processes are involved to an equal degree using the same task in different languages.

## Introduction

Reaction time experiments such as the word naming task are widely used to explore differences between L1 and L2 language processing. However, there continues to be debate about how to interpret naming latencies. It appears to be a major challenge to relate any of the many factors that are involved in the naming of a word (word frequency, word length, number of syllables, size of the neighborhood, the transparency of matching graphemes to phonemes, the pronunciation, and the degree to which word meaning plays a role) to a particular portion of the total naming time (de Groot et al., 2002). Even though the same factors can be assumed to be involved in naming a word on different occasions, the naming latency is subject to substantial variability, even when repeating the same word in succession (Kello et al., 2008) or when recording the naming latency of the same words on many different occasions (de Bot and Lowie, 2010). Since the components involved in the naming process flexibly, interactively and continuously contribute to a naming latency, the resulting naming latency is never an absolute constant.

Therefore, keeping in mind that the contribution of all the different factors involved in naming will be variable across tasks, languages, and participants, the current study aims at clarifying the degree to which automatic and controlled processing play a role in naming from an interaction-dominant perspective. Rather than trying to isolate the factors of interest in L1 and L2 naming, automatic grapheme-to-phoneme conversion and semantic involvement, the current study will use different tasks in which the relative contribution of these processes is assumed to be different. Analyzing the variability of naming latencies (RTs) during the task rather than the overall time it takes to name the words presents the opportunity to look at the relative contribution of automatic (grapheme-to-phoneme conversion) processes and more conscious, attention-demanding processes (semantic involvement) in L1 (Dutch) and L2 (English) word naming. With the possible exception of the most advanced speakers of an L2, word naming results consistently show language performance to be slower in an L2; however, longer naming latencies in the L2 do not necessarily indicate L2 naming to be qualitatively different from L1 naming (Segalowitz et al., 1999). Focusing on the variability of reaction times across a word naming task rather than an overall mean outcome can provide insight into the naming process as it is unfolding, and thus, into the nature of language processing.

A possible explanation why L2 language performance is usually slower in reaction time experiments could be simply the degree of use and practice. The underlying processing may be qualitatively similar in L1 and L2 language use in advanced L2 speakers, but due to less practice in using the language these processes could be less automatized and thus slower. However, another explanation could be that L1 and L2 language processing are qualitatively different, even for advanced L2 speakers. Using a language successfully requires the interaction of several components, such as mapping graphemes to phonemes, articulating sounds, and semantics. The relative contribution of these different components could be different between languages depending on proficiency and frequency of use. Looking at the speed of processing alone is not enough to distinguish between these accounts.

The central aim of the current study is to disclose the underlying processing involved in naming words in the L1 and L2, and to see if these processes are qualitatively different. A secondary question is if any differences that are found in the underlying processing, are attributable to the languages being a speaker’s L1 or L2, or are to do with differences between the languages. In order to answer these questions, the focus will be on the degree of automatization in naming words in an L1 and L2 as is shown from patterns of variability, and on assessing the relative contribution of semantic involvement in the naming process.

Since automatization and semantic involvement are part of language processing and take place over time, this study will use non-linear analyses to shed light on the underlying processes involved in naming. Originating in biology and ecology, but now also used extensively in other fields of cognitive science, spectral analysis is used to look at variability in response time data that has traditionally been regarded as irrelevant ‘noise’ but has been found to reveal the degree of automatization of mental processes (Van Orden et al., 2003; Kello et al., 2008; Wijnants et al., 2009; Lowie et al., 2014). Looking at patterns of variability in a word naming task in L1 and L2 allows us to look beyond the speed of processing at the difference in optimal coordination using the mother tongue or a second language.

The word naming task is a quick and undemanding task suitable for gathering language production data of multilingual speakers, without the drawback of confounding lexical processing with discrimination, as could be the case for the lexical decision task (Balota and Chumbley, 1984). For the purposes of the present article word naming also has the advantage of being continuous and can be applied without inter-experimental conditions, which allows for analyzing the set without interruptions and looking at processing over time. However, there is some debate concerning what processing the naming task actually taps into. Word naming is driven by orthography alone, in contrast to for instance picture naming, which is necessarily conceptually driven. In word naming, fast pronunciation is usually the only instruction; in this sense, semantic involvement could be seen as redundant with regards to the task goal. It is clear that semantic involvement is not a necessary requirement for pronunciation; one can pronounce a non-word or a word in a language unknown to the reader. Many dual route theorists indeed assume skilled readers to not use the semantic pathway when naming single words (Shibahara et al., 2003); however, semantic involvement has been found to play a role in naming low frequency, irregular words (de Groot et al., 2002; Shibahara et al., 2003). It thus remains unclear if, and to what degree, meaning plays a role in the pronunciation of single words. The amount of semantic involvement has also been found to vary between languages due to orthographic depth; that is, the transparency of a languages correspondence between phonology and orthography. The more complex a language’s spelling-to-sound conversions, the greater the orthographic depth (Katz and Feldman, 1983; Shibahara et al., 2003). There is some indication that for the multilingual speaker, reliance on semantics in reading varies for different languages with different orthographic depth, since more complex and inconsistent spelling-to-sound conversion rules may require semantic information in order to select a correct pronunciation (de Groot et al., 2002).

A word naming task is a suitable method for obtaining long, continuous data series in either the L1 or L2, and thus provides the opportunity to look at language processing over time. The different components (production and understanding) that contribute to natural language use are present, but a word naming task draws heavily on the production part of language use. In regular word naming, an automatic orthography-to-pronunciation link could be used in which semantics plays a minor role (de Groot, 2011, pp. 157–158). Due to a lot of practice, word pronunciation may be more automatized in a first than a second language. In order to test whether the underlying lexical processing is also more automatized in L1 naming than in L2 naming, a semantic categorization version of the naming task was devised, to ensure semantic processing during naming. The different versions of the naming task were tested on two different groups of participants; each participant was tested in the L1 and L2.

The resulting reaction time series of the word naming experiments were analyzed using spectral analysis, so as to gauge what happens over time when using the L1 or L2, rather than look at the product or result of the task. Spectral analysis can be used to look at the variability in the data, and gives an estimate of how random or structured the variability is. A structured pattern of variability is linked to more automaticity and a more optimal coordination of underlying processes. Of interest was the degree of automaticity in L1 and L2 language processing, and the difference between the standard word naming and the semantic categorization naming to assess the degree of semantic involvement in L1 and L2 language processing.

## Lexical Processing in Bilinguals

A consistent finding when comparing L1 to L2 naming is a speed difference; naming in the L2 has repeatedly been shown to be slower than naming in the L1 (e.g., experiment 3, Kroll and Stewart, 1994; La Heij et al., 1996; Van Wijnendaele and Brysbaert, 2002), even for fluent, (nearly) balanced bilinguals (Potter et al., 1984). These speed differences in L1 and L2 naming have been linked to automatic spelling–sound conversion and semantic involvement in naming, as in the 2002 study by Wijnendaele and Brysbaert. They found longer naming latencies for the L2 to be modulated by frequency; the difference in RTs was larger for low frequency words than for high frequency words, and more pronounced in L2 than in L1. The frequency effect is considered a marker of lexical involvement in the naming process, which leads the authors to conclude that the absence of a strong frequency effect in L1 naming suggests that L1 naming is largely mediated by automatized, non-lexical spelling–sound conversions, whereas the clear frequency effect found for naming in the L2 is taken to indicate that correct word naming in the L2 requires more lexical (whole word) mediation than word naming in L1, on account of there being a less powerful non-lexical route available for the L2 (Van Wijnendaele and Brysbaert, 2002). ERP studies, which are very sensitive to the time course of processing, seem to suggest that speed differences do not arise from differences in semantic processing. Several studies using event-related potentials (ERPs) have been conducted to look at differences in L1 and L2 processing. Even though these studies have found significant differences in syntactic L1 and L2 processing, semantic anomaly detection in sentence processing in high proficient L2 learners yielded very similar ERPs (Hahne, 2001; Hahne and Friederici, 2001) or a small delay of around 40 ms in L2 processing compared to L1 processing (Ardal et al., 1990).

de Groot et al. (2002) try to disentangle recognition and production as the locus of the effect of 18 variables. They compare lexical decision, standard word naming and delayed word naming to look at the “functional overlap” (Grainger and Jacobs, 1996) between the tasks and to see if a selection of 18 variables including word length, neighborhood, meaning, onset, frequency, etc. affected the processing of Dutch and English words in Dutch–English bilinguals differently. One of the most salient effects found by de Groot et al. (2002) is that standard naming and lexical decision responded very differently to the word manipulations, while the two tasks produced similar results in the L2 English. One of the language effects found by de Groot et al. (2002) most relevant to this study, is a semantic effect found for English naming but not for Dutch naming.

However, there is always a possibility that differences found between L1 and L2 language processing are confounded with differences that are actually attributable to differences between the languages themselves. Different languages may call for different processing strategies. An relevant example in the current context is the orthographic depth hypothesis (Katz and Feldman, 1983). The orthographic depth hypothesis concerns the differences between languages in the degree of complexity of their spelling–sound conversions. In the Katz and Feldman (1983) study, Serbo-Croatian and English naming are compared. They use Serbo-Croatian as an example of a language with shallow orthography; that is, very simple and direct spelling-to-sound correspondence. English, in contrast, has a deep orthography, with a complex, abstract correspondence between orthography and phonology. This leads the authors to hypothesize that readers of a shallower orthography may depend less on semantic mediation, and instead rely on fast, simplified spelling-to-sound correspondences. The absence of a semantic priming effect in Serbo-Croatian naming but not in English naming leads the authors to conclude that this is indeed the case. This effect was also found by Cuetos and Barbón (2006), who found semantic variables to affect Spanish word naming times less than orthographically deeper languages. For the present study, the orthographic depth hypothesis may also be relevant in explaining differences in Dutch and English naming; Dutch, like Serbo-Croatian and Spanish, has a shallow orthography with a more direct spelling-to-sound correspondence than English (van den Bosch et al., 1994). It is thus conceivable that in the naming of Dutch words, there also may be more of a reliance on fast and automatic spelling-to-sound conversion and less on semantic involvement.

Some research indicates at least reduced semantic involvement in word naming as compared to picture naming, lexical decision or translation (Lupker, 1984; Potter et al., 1984; Kroll and Stewart, 1994; de Groot et al., 2002). For instance, the poor recall of words in word naming as compared to picture naming has been interpreted as signaling reduced conceptual mediation and reduced depth of processing (Smith and Magee, 1980; Potter et al., 1984). de Groot et al. (2002) show that frequency effects are smaller in word naming than in lexical decision tasks, which is taken to be a sign of reduced involvement of semantic representations (de Groot et al., 2002). de Groot (2011, pp. 157–158) points out that for languages with an alphabetic script and regular grapheme–phoneme relations, the responses can be simple script-to-sound conversions, without any semantic processing. This would account for the difference found for the time it takes to name written words and pictures. It has been known for over a century that written words are named 200–300 ms faster than pictures (Cattell, 1886; Potter et al., 1984; Ries et al., 2015). The explanation for this difference in naming latencies is that a participant can start pronouncing a written word without the necessity of understanding it or retrieving the underlying concept. In this view, the process of naming consists of a series of stages that one has to go through to ultimately deliver the spoken word. In the case of naming a picture, the assumption is that the picture’s underlying concept first has to be found. This will make the identification of the correct word associated with this concept possible, and only then pronunciation can start. For written word naming, with fast pronunciation being the only goal, retrieving the underlying concept would be an essentially redundant stage that would only slow down performance, and pronunciation can start right away. An investigation into the time course of semantic and phonological encoding in a picture naming and a listening task using ERPs confirms the different order of semantic and phonological processing in different tasks and circumstances. A go/no go decision task based on phonological (first letter) or conceptual features (animal or object) showed that in picture naming, semantic processing preceded phonological processing by 170 ms. In listening, the effect was reversed, albeit smaller; phonological processing preceded semantic processing by 85 ms (Rodriguez-Fornells, 2002).

**FIGURE 1:**
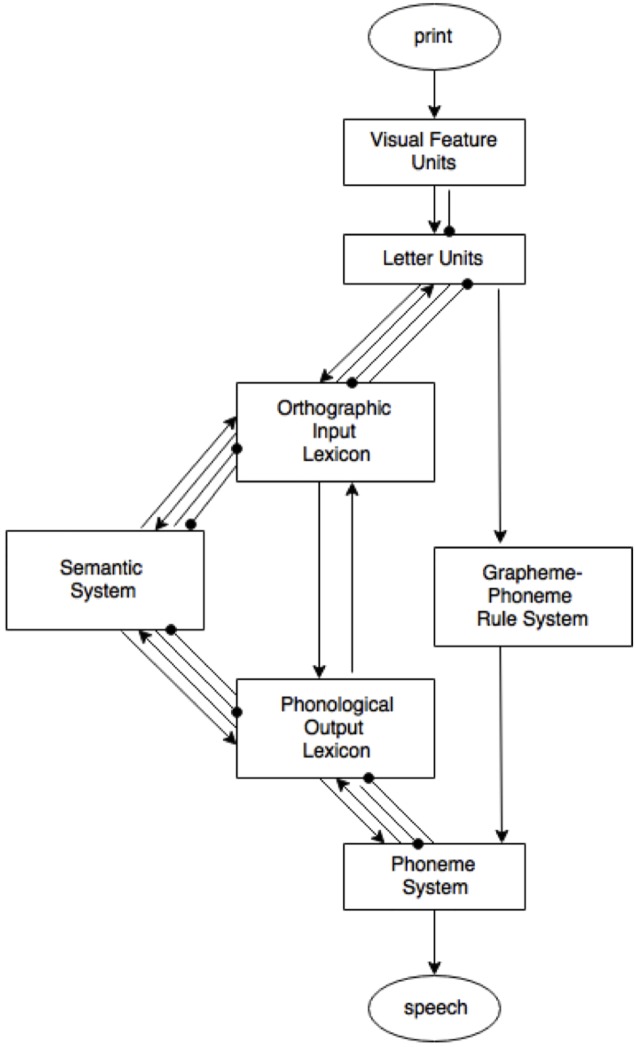
Dual-route cascaded (DRC) model. From “DRC: a dual route cascaded model of visual word recognition and reading aloud” by Coltheart et al. (2001). Copyright 2001 by the American Psychological Association.

Dual route models of word recognition visualize the stages from print to spoken word [dual-route cascaded (DRC) model (Coltheart et al., 2001) or the connectionist dual process (CDP+) model of reading aloud (Perry et al., 2007)]. **Figure 1** shows the DRC model (Coltheart et al., 2001), which is used here to visualize the processes that are potentially involved when naming a written word. This model depicts visual word recognition to be the product of the combined activity of an orthographic lexicon (the lexical route) and a rule-based grapheme–phoneme conversion (GPC) system (the non-lexical route). Word recognition in dual route models is based on visual encoding of orthographic information. The GPC system is necessary to account for the pronunciation of non-words or unknown words. Reading of (known) words makes use of both the lexical and non-lexical route; the difference being that through the lexical route, the pronunciation of the word is retrieved whole. After a lexical entry has been located in the orthographic lexicon, the matching phonology is accessed in the phonological lexicon. This information is then forwarded to the phoneme system, where the pronunciations of both routes comes together and the correct pronunciation can be chosen. Only use of the lexical route will result in the correct pronunciation of words with irregular grapheme-to-phoneme correspondence. Through the lexicon, the semantic system can be accessed. However, there are direct links between the lexicon and the phoneme system, which means that involvement of the semantic system is an optional ‘detour.’ Due to their modularity, dual route models (and most schematic models of language processing) feed into a dichotomous perspective on language processing; there either is semantic involvement, or there is not.

A possible alternative to dual models of word recognition is offered by parallel distributed processing (PDP) models, such as the PDP triangle model by Harm and Seidenberg (2004). The triangle model is a computational, connectionist model that aims to be biologically plausible, with a built-in ability to learn and self-organize. The 2004 model was focused on the access of word semantics directly through orthography, or indirectly, via phonology. This last way was envisaged as taking more time. Also in dual models of word recognition, when processing takes place through the lexical route, phonological information comes in relatively late, after the lexical item has been selected. Strong phonological models of visual word recognition make no distinction between a lexical and a non-lexical route; these models envisage phonology and semantics playing a major role in visual word recognition from the onset (Frost, 1998; Carreiras et al., 2014). In Frost’s computational model of word naming, phonology is always prelexically assembled and, if the task requires detailed phonology, lexically supported (lexical representations are needed, for instance, for the fully articulated, correct pronunciation of words with ambiguous spellings) (Frost, 1998). The model thus proposed by Frost is not dichotomous, but continuous, with both prelexical assembly and lexical/semantic involvement always playing a role in naming. This is in line with a study using a backward masking paradigm by Berent and Perfetti (1995), who find that the phonological representation of English CVC words is computed in two processing cycles with different time courses. In the first cycle, which is a fast and automatic prelexical process, consonants are computed. This generates a phonological skeleton of consonantal information. The second cycle is slower and involves attention-demanding processing; lexical information is used to fill in the vowels, which are the main source of lexical ambiguity (Berent and Perfetti, 1995). As Frost points out, if both lexical and prelexical computations affect the naming task, the focus of investigation should not be on whether there is lexical involvement on naming, but rather on the relative use of these processes in naming.

In models such as the ones discussed above, semantic representations are one of many components of stored lexical information. Lexical access in this view entails finding a simplified orthographic/phonological representation, whereas access to meaning is a later stage in word processing that can occur only after the lexical representation has been retrieved. The dissociation between lexical and semantic representations has long been taken as a necessary distinction to account for fast word processing. However, in the past decade this idea has been challenged. Elman points to the problem of where to draw the boundary between the lexicon and other types of linguistic knowledge. He found that many contextual factors (agent, tense, patient, location, filler of the instrument role, information given in the broader discourse context) influence expectations regarding the argument a verb will take. It would not do to list separate entries for every individual occurrence of a word, but any decision on what to include in the lexicon would also be rather arbitrary. He suggests “words do not have meaning, they are cues to meaning” (Elman, 2004, 2009, 2011). Spivey (2007) also contests the idea of discrete, static representations, and stresses the fluid nature of cognitive processing, claiming that mental (whether lexical, semantic, or otherwise) representations should be thought of as processes: “sparsely distributed patterns of neural activation that change non-linearly over the course of several hundred milliseconds, and then blend right into the next one” (Spivey, 2007). Applying these findings to the current study, it would follow that it is not necessarily a fruitful path to follow to think of a sequence of events as pre-lexical (e.g., phonological assembly) or post-lexical (e.g., semantic access), or to think of semantics and meaning as being mediated by lexical entries in a mental lexicon, nor would it be interesting to see whether or not semantics plays a role in word naming. Assuming the activation of meaning to be a gradual process that plays a role in word recognition, but perhaps to a different degree depending on for instance language dominance, the focus of interest would then be the relative contribution of meaning activation in naming.

Models such as the dual route models of word recognition do not specify if and how the word recognition process would be modulated for multilingual language use. A lot of research has focused on differences between L1 and L2 language processing, such as the influence of the L1 on L2 processing, but as Lemhöfer et al. (2008) point out, these studies have been conducted with carefully selected and few test items, with the aim of uncovering differences. Whether or not these differences would still exert an influence in natural language processing is uncertain; after all, in less controlled conditions it may be balanced out by many other factors playing a role in processing. Lemhöfer et al. (2008) conducted a study of bilinguals with different L1’s (French, German, and Dutch) to look into the L1 specific influence on L2 processing. They used a multiple regression model that allowed them to look at the influence of many variables, including word frequency and length, concreteness and meaningfulness and many others. Apart from cognates, L1 specific influence on L2 processing was absent, and they conclude that L2 processing is “language driven,” meaning that within-language factors seem to determine the adopted processing strategies. However, even though bilinguals with different L1 backgrounds process the L2 in the same way, they did find differences in the language processing of native speakers and L2 speakers, especially on word variables related to frequency and ways of occurrence. These variables exert a much greater influence on L2 processing than on L1 processing, and the authors conclude that L2 processing is thus “fundamentally different” from L1 word processing (Lemhöfer et al., 2008, p. 27).

That L1 and L2 language processing are different, has been made visible by a range of neuroimaging studies. A marked difference is the level of attention or control needed for bilingual language processing. In an fMRI study looking at age of acquisition and proficiency effects in L1 and L2 processing, Wartenburger et al. (2003) found more extensive cerebral activation during semantic judgment tasks for L2 processing than L1 processing. In a set of studies using EEG and fMRI, Rodriguez-Fornells et al. (2002) compare monolingual and bilingual language processing and found more left prefrontal activation in the bilinguals, associated with increased control and language inhibition. Important factors that modulate differences in bilingual versus monolingual language production are relative proficiency in each language, as well as the level of competition between both languages (Abutalebi and Green, 2007; Kroll et al., 2008; Green and Abutalebi, 2013). Meschyan and Hernandez (2006) show in an fMRI study differential processing in languages that differ in orthographic depth; Spanish is a very transparent language, and English has a deeper orthography. They found that different brain regions were involved when English–Spanish bilinguals were reading Spanish words than when reading English words. The study shows the bilingual adapting to the most optimal lexical access strategies of the target language; a skill that is likely to develop with increasing proficiency (Meschyan and Hernandez, 2006). In an overview study looking at results of a large number of fMRI and PET studies that investigate bilingual language production, Abutalebi and Green (2007) argue that an increase in proficiency is accompanied by a shift from controlled to more automatic processing, and by a reduction in prefrontal activity. Increasing proficiency will lead to a dynamic adaptation of the language system and the control networks. However, the authors point out that it is likely that the bilingual will not process the L2 identical to the way a monolingual speaker of that language would. In the same vein, they hypothesize the bilingual’s processing of the L1 is unlikely to be identical to a monolingual’s processing of the L1.

### Interaction Dominance and Self-Organized Criticality in Language Processing

An essential question to pose before embarking on research into language processing and the role of semantics in processing, is the question what kind of underlying system it is we are confronting. In cognitive science, including language research, the computer metaphor of the mind has been hugely influential. The human mind being considered as too complex to study as a whole, it was decomposed into different parts that could be studied separately, giving rise to a modular view of the mind (Fodor, 1983). Another way of describing this view is the *component dominant* view. In this view, any changes in the system can be traced back to isolated, independent information-processing modules, as in the dual-route models mentioned above. This view has inspired a lot of empirical studies and has generated many (modular) models, and produced many insights. However, when it comes to objects of study that are very complex, consisting of many interacting variables, as is the case in biological and psychological systems, a modular and linear model can also lead to oversimplification. When many variables continuously interact, trying to isolate them in a study can produce mixed results and studies that are hard to replicate. In the language system, as well as the cognitive system as a whole, isolating components that contribute to change or development proves to be very difficult. Human behavior is often the result of many interacting factors and is very hard to explain by one or several separate factors.

An alternative to the component-dominant approach of cognition is offered by *interaction-dominant dynamics*. In interaction-dominant dynamics, the behavior of the system emerges from the non-linear interaction between many interdependent components that are nested within each other (Gilden, 2001; Van Orden et al., 2003; Spivey and Dale, 2004; Kello et al., 2008). This non-linear interaction and nested entanglement makes it impossible to look at the contribution of one particular, isolated component to a task or process. Spivey and Dale (2004), for instance, show how visual and verbal behavior have often incorrectly been treated as functioning separately, rather than interacting continuously. Components in the system do not only interact across domains, such as visual and verbal clues, but also within. Many different measures of intrinsic variability in human behavior show a type of variability that is indicative of these non-linear interactions called 1/f noise or pink noise (Kello et al., 2007). Pink noise is a type of variability that is temporally related; there are both short and long range correlations. This is opposed to the white noise that is assumed when linear statistics are used, which is the uncorrelated, random variation around a mean. Gilden (2001) showed non-linear, interaction-dominant dynamics, to govern behavior in a wide variety of tasks, from visual search to lexical decision.

Where white noise is associated with random behavior and does not show correlation between measurements, brown noise is associated with over-regular behavior, and shows very strong dependence between measurements. Pink noise can be found in between white noise and brown noise, between over-random and over-regular, and can be observed when there is a balance between the two (Gabbay, 2010). Rigid, over-regular control only works in a very predictable environment, but fails when the environment becomes less predictable; over-random performance allows for flexible behavior, but cannot take advantage of the predictable features of the environment. A system that is in balance between white and brown noise thus allows for an “optimal combination between stability and flexibility in control” (Van Orden et al., 2009, p. 30). Interaction-dominant dynamics thus offer an explanation for the soft assembled, context sensitive nature of cognition and language processing.

There is plenty of proof that cognition and language use are governed by interaction-dominant dynamics that produce this correlated pattern of variability. Response times series, which are widely used in psycholinguistics as evidence for models of lexical processing, categorization and decision making, elicit a pattern of variability that is not random but shows pink noise, e.g., lexical decision (Gilden, 1997), visual search (Gilden, 2001), simple reaction times (Van Orden et al., 2003), and word naming (Van Orden et al., 2003; Wijnants et al., 2012; Lowie et al., 2014). An example is a study by Van Orden et al. (2003), where participants took part in a simple reaction time experiment and a word naming experiment. The simple reaction time experiment required participants to repeat /ta/ into a microphone every time a signal to respond (#######) appeared on the screen. This simple reaction time experiment consisted of 1,100 trials and yielded spectral slopes ranging from -1.00 to -0.30, with a mean of -0.66. This is consistent with the scaling exponent of pink noise. The word naming task, which is more representative of actual language use than the simple reaction time experiment, consisted of 1,100 monosyllabic words in a unique random order. Spectral analyses generated spectral slopes ranging from -0.49 to -0.14, with a mean of -0.29, that were found to be consistent with pink noise (and reliably different from slopes generated by the same randomized data). Van Orden et al. (2003) posit that the difference found in the steepness of slopes can be explained by random word properties decorrelating the dependency relation between the sequence of responses over time in the naming task. A noise signal that shows fewer long-term correlations is closer to random, white noise, and thus farther removed from the pink noise that is associated with optimal coordination of subsystems involved in processing. Another study that finds clear pink noise in a language production task is Kello et al. (2008) in analyzing the acoustic signal of 1.100 repetitions of the word “bucket.” Variability in the acoustic measurements was found to show near ideal 1/f scaling of 1.06, a scaling typically associated with a system that is near a critical state (Kello et al., 2008).

A critical state is a state in the system that allows for two opposing options in behavior. In order for this balance between different options to be maintained, critical states function as attractor states. The construct that critical points are attractors in complex systems is called self-organized criticality (SOC), (Bak et al., 1987). There is evidence that pink noise is a by-product of an attractor state, since development and training have been found to change behavior that elicits random white noise to more pink noise (Wijnants et al., 2009). Wijnants et al. (2009) conducted a precision aiming task where participants had to draw lines as fast as possible between two dots that were 24 cm. apart using their non-dominant hand. The idea was that forcing participants to use their non-dominant hand would induce relatively unstable and uncoordinated behavior that would leave plenty of room for improvement. Participants would complete five blocks of 1,100 trials with 3-min breaks in between the blocks. The time it took to trace from one dot to another was measured. Trace times on the early blocks were found to be quite random and show a scaling exponent around 0, consistent with white noise and thus irregular and uncoordinated behavior. With practice, the trace times of the later blocks show scaling exponents that approach -1, the scaling exponent of pink noise (Wijnants et al., 2009). This trend toward pink noise can be seen as attraction toward pink noise (Van Orden et al., 2009).

The idea that practice and development lead to a system being more optimally organized and thus exhibiting pink noise has been applied to language tasks in the L1 and L2. In a longitudinal study carried out by Lowie et al. (2014) one very proficient subject was tested over an extended period of time, during which the amount of usage and exposure to the L1 and L2 was varied. After a stay in exclusively the L1 or L2 language environment, the pink noise scaling relation was significantly stronger for the language recently used. This implies that after a period of exclusively using one language, the underlying system is more stable and optimally coordinated for that language. For a language learner not staying in an L2 environment, speaking in the L2 may be more like using the non-dominant hand in the tracing task mentioned above; due to less practice, L2 usage could be relatively unstable and uncoordinated. For participants using their L1 in their L1 environment the underlying system is expected to be stable and optimally coordinated; however, when using the non-dominant language in the L1 environment, there will probably be a less optimal coordination of subsystems and hence a scaling exponent closer to white, random variability.

### The Present Study

The different factors involved in language processing have been found to be numerous, and their contribution to the processing either inconclusive or inconsistent between languages and across individuals and different moments of testing. Therefore, rather than looking at the exact contribution of factors involved in language processing at any specific moment in time, it is more useful to look at the relative contribution of factors involved in language processing over time. Taking into account the complex, variable and adaptable nature of language processing, the current study takes a processing perspective to look at the relative contribution over time of two major factors (automatic grapheme-to-phoneme conversion and attention-demanding semantic processing) in reading out loud. The results will provide insight in the contribution of these factors in L1 and L2 language processing, and will provide an answer as to whether or not L1 and L2 processing are qualitatively different.

The discussion on the role of meaning in L1 and L2 language processing thus far has been rather dichotomous. Semantic involvement has been found to play a more limited role in single word naming than in other experimental paradigms such as lexical decision or picture naming. The dual route processing models feed into this dichotomous discussion, by allowing processing to travel along a ‘semantic’ route, or to bypass semantics altogether. de Groot et al. (2002) found a semantic effect for English L2 naming but not Dutch L1 naming. It has also been suggested that semantics play a more prominent role in naming low frequency, irregular words (Van Wijnendaele and Brysbaert, 2002), and that this effect is stronger in an L2, which makes sense, considering that L2 words are less frequent for L1 dominant L2 speakers. Rather than trying to answer the question of whether or not there is any semantic involvement in L1 or L2 word naming, this study will use non-linear analyses to determine whether the relative degree of conscious, semantic involvement and automatic spelling-to-sound conversion processes differs when naming in an L1 or an L2.

In order to determine whether semantic involvement plays any role in regular word naming, two versions of a word naming experiment were devised. One regular version in the L1 and L2, the only instruction of which was to name the words correctly, and as fast as possible. The other, semantic condition version of the exact same experiment had the added instruction of the participant being required to press a button whenever the word appearing on the screen denoted an animal. The resulting response time series were analyzed using spectral analysis, which is a type of analysis that yields a slope line. The steepness of this line gives information about the pattern of variability in the time series; a slope line of zero indicates a random pattern of variability, whereas a slope line of -1 indicates pink noise, or a fractal pattern of variability. A pink noise pattern indicates optimal coordination and a high degree of automatization in the sub-processes contributing to a task (Van Orden et al., 2003; Wijnants et al., 2009, 2012).

Comparing the slope statistics of standard L1 word naming to standard L2 word naming will show if the degree of automatization in language processing is different in L1 and L2 naming. Since the L1 is the dominant language for the participants, subcomponents contributing to language output are expected to be more tightly coupled and be more efficiently and optimally coordinated. Therefore, slope statistics closer to the scaling exponent of pink noise are expected in the Dutch L1 standard naming condition. The L1 and L2 semantic naming condition are compared to gain insight into the relative degree of automatic vs. attention-demanding semantic processing in L1 and L2 word naming. More attention-demanding semantic processing is expected to decorrelate the variability pattern, and yield slopes that are closer to random variability. Of interest is whether processing in both languages is affected to an equal degree, which will give information about the relative degree of automatic vs. attention-demanding processes in L1 and L2 word naming.

There is one other factor that will be considered in this study. Since the studies mentioned above (de Groot et al., 2002; Van Wijnendaele and Brysbaert, 2002) that have looked at semantic involvement in L1 and L2 processing both focus on L1 Dutch and L2 English, the observed differences in semantic involvement could be due to the languages under investigation rather than their L1 or L2 status. A relevant difference between Dutch and English is the orthographic depth; Dutch being a language with more transparent grapheme-to-phoneme correspondences than English could account for the language allowing more automatic spelling-to-sound conversion. In order to distinguish between a language effect or an L2 effect, the data obtained in this study will be compared to the results from an earlier study conducted by Van Orden et al. (2003) that reports on L1 English word naming data and reports spectral slopes that can be directly compared to the L1 Dutch and L2 English slope statistics collected here. Comparing the L1 Dutch naming and L2 English naming data obtained here to the Van Orden et al. (2003) data will allow us to distinguish between a language effect or an L2 effect. If the spectral slopes found for the L1 English naming condition differ from the L2 English naming data, we can conclude that this is likely due to an L2 effect. If the steepness of the spectral slopes observed in the L1 English naming data differ from the steepness of the slopes found for the L1 Dutch naming data, it is likely there is a language effect.

### Analysis

The analysis used here to distinguish a fractal structure in the data (coordinated behavior) from random variation is spectral analysis. Spectral analysis can be used to look at the variation in a signal. It transforms a data series from the time domain (milliseconds) into the frequency domain (Hz) through a Fast-Fourier-Transformation (for an explanation, see Wijnants et al., 2009). Spectral analysis finds the best fitting sum of sine and cosine waves of the time series, and plots their amplitudes and frequencies on log–log scales. The resulting plot shows the relation between amplitude and frequency. To interpret whether the time series shows random variability or some structure/regularity, a line is fitted to this plot. If the slope of this line is zero this indicates random variation, while a slope of -1 indicates pink noise. An even steeper slope of -2 (brown noise) will indicate an even more regular structure of variation in the original time series.

To further determine whether the slope statistics obtained from the spectral analysis deviate reliably from white noise, the original, time-ordered data can be randomized. If the order in which the data points are collected is disturbed, finding the short and long range correlations that indicate a fractal pattern or pink noise would not make sense, and one would expect to find a zero slope line indicating an absence of correlations (and thus: dependency between succeeding data points) and random variation when analyzing the randomized data.

To conduct a spectral analysis, some preprocessing of the raw data is required (Holden, 2005). Extreme values may skew the results, so values below 100 ms and above 1,000 ms were removed. Any remaining outliers above and below 3 SDs were removed. The data was then detrended. Spectral analysis requires a number of data points that is a power of 2, so the data was then truncated to 512 data points. Data points were cut from the beginning of the time series.

## Materials and Methods

### Participants

Participants (*N* = 42) were students of the English Language and Culture BA program at the University of Groningen in the Netherlands. Most of them were female (*F* = 32, *M* = 10). Ages ranged from 19 to 43 years (mean 24 years, median 22 years). Participation was a course requirement. Participants were asked for information on language and academic background. All participants were native Dutch speakers with near-native English proficiency (CEFR level = C2). In the academic context, these students almost exclusively use English; all classes, assignments and course materials in this BA program are in English. The participants in this study had started learning English as a foreign language in formal education between the ages of 10 and 13 years old. Dutch is the dominant language in the Netherlands in everyday life. Even so, English acquisition may have started earlier due to the presence of the English language in the Netherlands in media, music, internet, and games (for a more elaborate discussion of the position of English in the Netherlands, see Gerritsen et al., 2016). None of the participants had reading disabilities. All had normal or corrected-to-normal vision.

### Stimuli

Stimuli were 550 monosyllabic English words and 550 monosyllabic Dutch words, selected using CELEX (Baayen et al., 1995). Word length was 3, 4, or 5 letters. All words were high frequent concrete nouns with simple (CV) onsets, making them easy to pronounce. English–Dutch cognates, homographs and homophones were avoided. Each language set contained 50 words denoting an animal.

### Apparatus

Stimuli were presented on a 17″ monitor. Stimuli were presented using the E-Prime software (Psychology Software Tools, Pittsburgh, PA, United States) (Psychology Software Tools, Inc., 2006). Response times were measured up to the point where the participant would start to pronounce the word on the screen, and were recorded by E-Prime. The responses were recorded using a portable voice recorder. Error responses, where the voice key had been triggered by breathing, coughing, or swallowing noises or where it failed to record because the spoken response was too soft were excluded.

### Procedure

**FIGURE 2:**
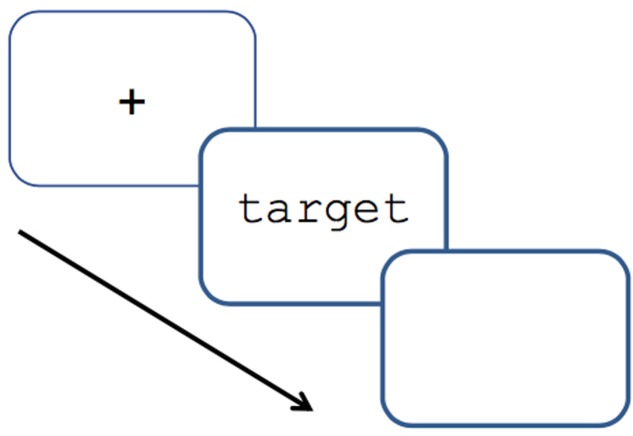
Procedure of the presentation of targets.

Participants were tested individually in a soundproof room. The English and Dutch version of the experiment were conducted consecutively. Half the participants started with the Dutch language version, the others with the English language version. Participants were randomly assigned to the standard or the semantic condition. The button that had to be pressed in the semantic condition was a red button in a black box, connected to the computer with a USB cable; button presses were not recorded. The participant sat facing a computer screen, with a microphone placed on the table in front of him. The test items appeared in black, lowercase Courier New (24 points) on a white background. The experiment started with an instruction slide informing the participant to pronounce the words appearing on the screen as quickly and accurately as possible. In the semantic condition, instructions were the same, with the added instruction to press a red button if the word appearing on the screen denoted an animal. Each target was preceded by a fixation point in the middle of the screen for 500 ms. The target would appear on the screen, and remain there for maximally 5,000 ms, or until a response was recorded. The target would remain on the screen for 300 ms after the participant had started to pronounce the word, to ensure that it was still on the screen during the pronunciation. The screen would be blank for 1,000 ms, after which the next fixation point would appear. The procedure is visually represented in **Figure 2**. The order of presentation of the test items was randomized automatically by the computer program for each participant. A practice block of 10 items preceded the experimental block to familiarize participants with the task requirements.

## Results

### Means Analysis and Results

The results are summarized in **Table 1**. If the voice key was set off by accident or if no response was recorded within 1,000 ms, the data point was counted as an error and removed before any analyses were carried out.

**Table 1:** Mean RTs, SDs and percentage of errors of the naming task in Dutch (L1) and English (L2) in the standard condition and the semantic condition (SC).

	SC L1	SC L2	L1	L2
RTs	503 ms	533 ms	505 ms	545 ms
SDs	93	101	70	84
Errors (%)	1,4	3	1	1,4

The data were analyzed using a repeated measures ANOVA. Results are summarized in **Table 1**. Mean RTs on the Dutch sessions are faster than on the English sessions. The effect of language is found to be significant, *F*(1,19) = 57.363, *p* < 0.001. There was no significant effect found for the semantic vs. the standard condition, and the interaction between naming condition and language was also found not to be significant.

### Spectral Analysis and Results

Spectral analyses were carried out to establish whether, regardless of how fast or slow the naming performance, naming is more automatized and optimally coordinated in the L1 than in the L2, and whether the stronger role of semantics in the semantic categorization condition would lead to less reliance on automatic grapheme-to-phoneme conversion and more attention-demanding semantic processing in naming. Spectral analyses were run on all individual sessions, as well as on the aggregate data of each of the conditions.

Before the spectral analyses were carried out, the data was truncated to 512 data points. On average, 35 data points were removed from the beginning of the time series. Linear trends were removed (see Holden, 2005, p. 288) and the data were normalized.

For each of the time series, 64 frequencies were calculated. To estimate the spectral slope, a slope line was fit to 50% of the frequencies. Spectral slopes found for L1 Dutch standard naming were steepest, showing the strongest fractal pattern, ranging from -0.58 to 0.04, *M* = -0.38. Spectral slopes for L2 English standard naming ranged from -0.47 to 0.04, *M* = -0.19. Spectral slopes found for L1 Dutch naming in the semantic condition ranged from -0.50 to 0.00, *M* = -0.28, spectral slopes for L2 English naming in the semantic condition ranged from -0.40 to 0.00, *M* = -0.19. **Table 2** shows the mean spectral slopes for each of the conditions.

**Table 2:** Mean spectral slopes (SSs), SDs and percentage of errors of the naming task in Dutch (L1) and English (L2) in the standard condition and the semantic condition (SC).

	SC L1	SC L2	L1	L2
SSs	-0.28	-0.19	-0.38	-0.19
SDs	-0.13	-0.10	-0.18	-0.18
Errors (%)	1,4	3	1	1,4

**FIGURE 3:**
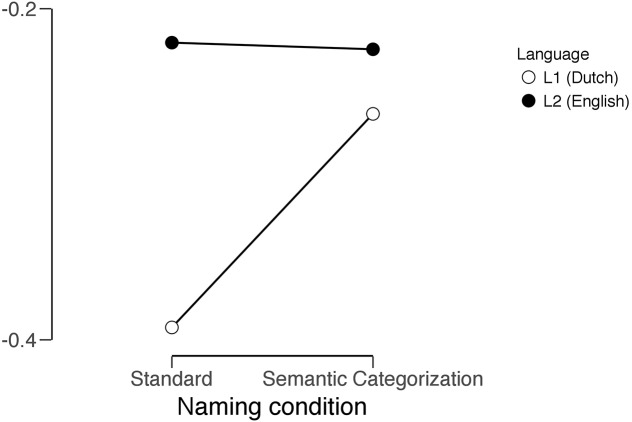
Graphical representation of the interaction between language and naming condition. Spectral slope statistics on the *y*-axis, naming condition on the *x*-axis.

Again, the data were analyzed using a repeated measures ANOVA. As is the case for the RTs analysis, the main effect of language was significant, *F*(1,19) = 13.189, *p* = 0.002. In comparing the semantic vs. standard naming condition no significant effect was found for the main effect of condition; however, the interaction between naming condition and language was found to be significant, *F*(1,19) = 4.857, *p* < 0.04, showing that there was a larger decrease of the spectral slopes for L1 compared to L2. **Figure 3** graphically depicts this interaction.

**FIGURE 4:**
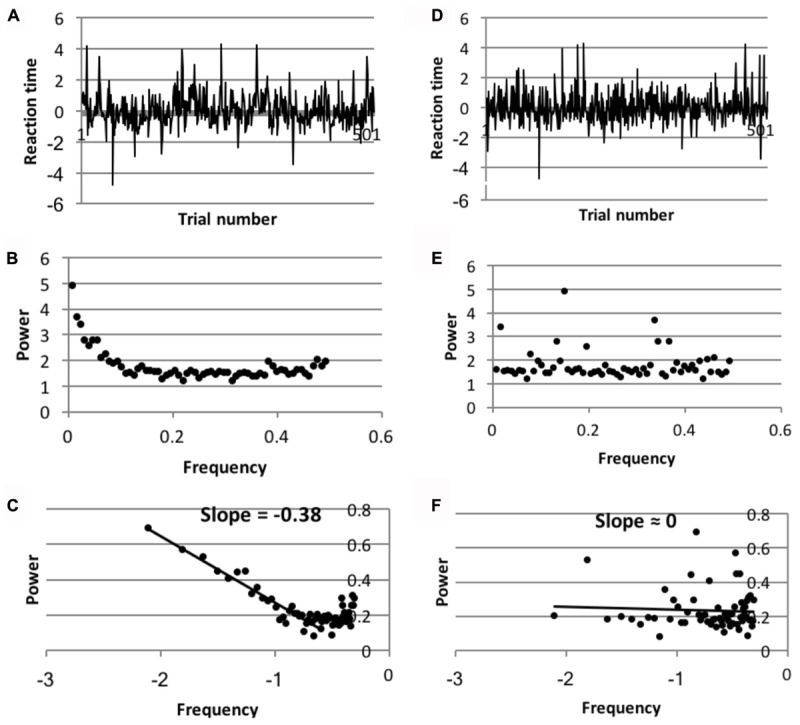
**(A–C)** The spectral analysis of the intact trial series of the aggregate data of the Dutch language trial series in the non-semantic condition. **(D–F)** The same analysis of the randomized data. **(A,D)** Detrended and normalized reaction time trial series of one of the Dutch participants; **(B,E)** simple reaction time power spectrum of the aggregate data in the Dutch standard naming condition; **(C,F)** power spectrum on log–log scales of the aggregate data in the Dutch standard naming condition.

To test the validity of the spectral slopes found on all conditions, data was compared to randomized versions of the same data, but with the time ordered sequence of the data disrupted. Spectral slopes on all conditions were found to differ significantly from slopes fit to the randomized data. **Figure 4** shows this comparison for the aggregate data of the Dutch standard naming data.

## Discussion: Automatization and the Degree of Lexical Involvement in Naming

The present study aimed to investigate the relative contribution of automatic processes (grapheme-to-phoneme conversion) and attention-demanding processes (semantic involvement) in L1 and L2 language production using a standard word naming task and one that required semantic categorization. Spectral analysis was used to look at the degree to which the L1 and L2 language subsystems were optimally coordinated and automatized during word naming, and the degree to which semantic processing played a role in word naming.

A fractal pattern of variability was found for both L1 Dutch and L2 English word naming. The spectral slopes found for L1 Dutch standard word naming (-0.38) were significantly steeper than the spectral slopes found for the English standard naming condition (-0.19). It is important to stress that the steepness of the spectral slopes is not related to the speed of processing; even though L2 naming is consistently slower, this does not lead to a relative difference in the steepness of spectral slopes. Speed of processing and scaling are independent of one another. As indicated, a fractal pattern of variability is associated with optimal coordination and automatization of the mental subsystems contributing to a task. The fractal structure in the L2 data shows a more decorrelated pattern closer to random variation. The stronger fractal pattern in the L1 data indicates a more optimal coordination of mental subsystems when naming L1 words than when naming L2 words. In other words, the appearance of a string of letters on the screen that correspond to an L1 word leads to a highly automatized, smooth response that is consistent with an account of automatic spelling-to-sound conversion in the L1. The same degree of automatization does not seem available in the L2.

The same naming task in the semantic condition was conducted to ensure that the processing that was being investigated was not only governed by automatic spelling-to-sound-conversion, but also semantic processing. Comparing this task to the standard word naming allows us to infer the degree of semantic processing present in standard word naming; if no difference between the conditions were found, the same degree of semantic processing must already be present in standard word naming. However, the semantic categorization condition did have a differentiating effect in the L1 and L2; a comparison of the L1 standard naming condition with the L1 semantic naming condition shows a more decorrelated fractal pattern of variability in the L1 semantic naming condition (-0.38 for L1 standard naming and -0.28 for L1 semantic naming). The same effect was not found in L2 naming; both standard L2 naming and L2 semantic naming generated the same mean spectral slope of -0.19. This finding is in line with the observation that L1 naming relies more than L2 naming on automatic spelling-to-sound conversion. The semantic categorization condition would have forced more attention-demanding lexical involvement than regularly used in the reading aloud of L1 words, leading to a variability pattern that was less fractal than the one observed for L1 standard naming. If automatic spelling-to-sound conversion is less available in the L2, as suggested by de Groot et al. (2002) then semantic/lexical involvement already plays a significant part in L2 naming, and the manipulation would not have made a difference to the normal procedure of reading aloud single L2 words.

**FIGURE 5:**
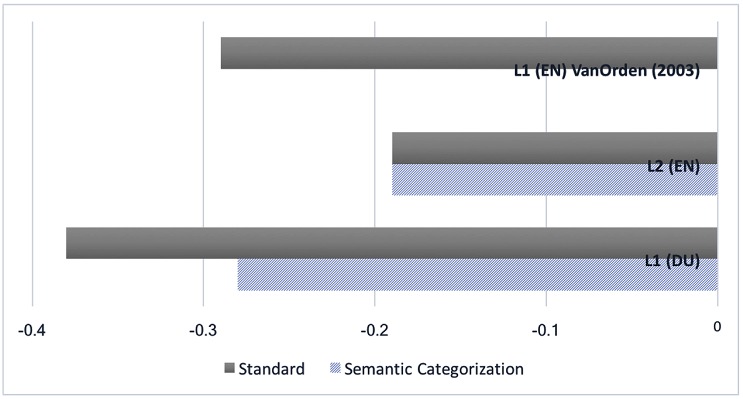
Comparison of the mean spectral slopes of the standard and semantic naming condition in the L1 Dutch and L2 English to the mean spectral slopes of L1 English standard naming obtained by Van Orden et al. (2003).

To find out whether the observed difference in slope statistics comes down to a difference between L1 and L2 processing, or between Dutch and English language processing (recall the orthographic depth hypothesis) it is useful to compare the languages in the semantic categorization condition, and to also compare these results to L1 English standard naming, as obtained by Van Orden et al. (2003)^1^. For easier reference, the **Figure 5** combines the results obtained in the present study, and those found by Van Orden et al. (2003).

The spectral slope found for the Dutch semantic condition was less steep than the slope found in the standard naming condition, but at -0.28, it was still steeper than the spectral slope of -0.19 found for the English semantic condition. So, even if the degree of lexical involvement is manipulated for both languages, a language effect remains intact, indicating more automatized processing in the L1. However, these results do support the orthographic depth hypothesis, which classifies English as having a deep orthography with an inconsistent spelling-to-sound correspondence that requires lexical mediation for correct pronunciation. The slope of -0.28 found for the L1 Dutch semantic condition corresponds almost exactly to the mean spectral slope of -0.29 reported by Van Orden et al. (2003) for English L1 standard word naming (Van Orden et al., 2003). The differences in the relative contribution of automatic processes (grapheme-to-phoneme conversion) and attention-demanding processes (semantic involvement) in L1 and L2 language use found in this study seem to be attributable to two different causes. The steeper slopes in L1 standard Dutch naming (-0.38) than in L1 standard English naming (Van Orden et al., 2003) can then be attributed to the difference in orthographic depth of the languages; Dutch having the shallower orthography, allows for more automatic spelling-to-sound conversion. However, the L2 status of English for the participants in this study did still generate an effect beyond the deeper orthography, participants using more attention-demanding processing in the L2 than the English native speakers in the Van Orden et al. (2003) study, as reflected in spectral slopes of -0.19.

This leads to another observation on account of the advanced L2 learners participating in the current study. Even though they do not achieve the optimal coordination of underlying subsystems when using the L2, they are adopting language processing strategies with a higher relative contribution of lexical mediation than is usual in their L1, thus adopting native-like language processing strategies, albeit less optimally organized than native speakers.

## Conclusion

The goal of this investigation was to look at the relative contribution of automatic grapheme to phoneme conversion processes and more conscious, attention-demanding processes (lexical/semantic involvement) in L1 Dutch and L2 English word naming. As hypothesized, the relative contribution of automatic vs. attention-demanding processes in the L1 and L2 was not equivalent, even though the learners participating in this study were near-native L2 speakers. The scaling relation found for L1 naming as compared to L2 naming was closer to the scaling relation indicating automatic, optimal coordination of mental processes, probably achieved through more regular use and practice of the L1. This conclusion is in line with Lowie et al. (2014), who looked at a single native Dutch speaker who was very proficient in the L2 and regularly spent time in the L2 environment; they found steeper spectral slopes for the language that was recently used, regardless of it being the L1 or L2, and a decorrelated fractal structure for the language that was not recently used.

In addition to, and related to the relative contribution of automatic spelling-to-sound conversion, the focus of interest was the relative contribution of more attention-demanding, semantic processing. The semantic condition was found to affect patterns of variability in the L1 only, leading to the conclusion that L2 speakers rely more on lexical involvement in naming words. Even when forcing more contribution of semantic processing in the L1, the spectral slopes still indicated a higher contribution of automatic spelling-to-sound conversion processes in L1 naming than in L2 naming. We would therefore like to propose an amendment to the conclusion reached by Lemhöfer et al. (2008), who conclude that L2 processing is mainly language driven. The present findings with a group of very proficient bilinguals, would lead us to the conclusion that it is in part language driven, and determined by the language properties such as orthography of the target language. However, language use also is also user driven, determined by the bilingual language system, including factors such as frequency of use that constrain the language production process and lead to more controlled processing.

This study is not the first to find L2 processing is less automatized than L1 processing (Rodriguez-Fornells et al., 2002; Wartenburger et al., 2003; Abutalebi and Green, 2007; Green and Abutalebi, 2013). However, by looking at the relative contribution of automatic and controlled processing from an interaction-dominant perspective, it offers an explanation how and why this might be the case. From an interaction-dominant perspective, in which there is a coupling and coordination of contributing subsystems, different languages within a multilingual speaker being more or less automatized becomes explicable, and it becomes possible to examine exactly which processes contribute to making the coordination of underlying subsystems more automatic or controlled, in this case by manipulating the degree of semantic involvement in the task. The dynamic approach used here shows a quantifiable, testable way of looking into the self-organizing behavior of the language system, and how it constantly adapts, both to language specific factors such as a language’s orthographic properties, and to the language user’s capabilities and constraints related to the frequency of use of a language.

This differential degree of automatized and controlled processing in L1 and L2 naming has implications for how to interpret the extensive body of research into multilingualism that uses this task to look at word processing differences between languages in multilinguals. Earlier research already indicated care should be taken in interpreting evidence on word recognition that may be confounded with task-specific processing (Balota and Chumbley, 1990; de Groot et al., 2002), since for instance a lexical decision task may tap into different processing strategies than a word naming task. However, the results from the current study show that even using the same task in different languages may tap into different processing strategies. Related to this, and pointed out by Abutalebi and Green (2007), is that when the system adapts toward strategies that are most appropriate to process the L2, the entire system changes. Therefore, increasing proficiency in optimally processing the L2, and specifically L2 learners using semantic processing more in the L2 than they are used to in the L1, could change the way in which the L1 is processed as well. For the current study, this means that it would be interesting to compare the results to monolingual Dutch speakers as well, since the participants in the current study possess a language system adapted to processing an L2. Furthermore, whereas the current study shows semantic reliance to differ between languages within an individual, different individuals also differ in the degree of semantic involvement used in word naming (Woollams et al., 2016).

These results taken together indicate caution is needed when interpreting studies that are generalized over individuals, monolinguals and bilinguals, languages or tasks. If word naming in the L2 tests ‘deeper’ processing than naming in the L1, caution is needed when comparing the results of multilingual naming. When a task such as word naming is used in bilingual research, the aim is to be able to generalize about a variable that has been manipulated. The assumption is that apart from this manipulated variable, the same task in different languages is testing the same underlying processes. The (multilingual) individual’s adaptability to task, circumstance and environment is underestimated in such direct comparisons; this study suggests caution is needed when comparing cross-linguistic results of word naming tasks.

## Ethics Statement

The study was exempt from ethical considerations. The experiment concerned a non-invasive reaction time experiment. Participants were adult, healthy university students, who participated voluntarily and received a small reward. For some participants, participation was part of course credit.

## Author Contributions

RP, principal author, carried out the study, analyzed the results, and wrote most of the paper. WL, promotor and daily advisor, has been involved in designing the study and providing advice and feedback along the way. KdB, co-promotor, was involved in the design of the study and giving advice and also offering feedback during the writing of the paper.

## Conflict of Interest Statement

The authors declare that the research was conducted in the absence of any commercial or financial relationships that could be construed as a potential conflict of interest.

## Footnotes

1 No language background information is available on the Van Orden et al. (2003) English L1 speakers, so it is unknown whether they were proficient in additional languages. Without information to the contrary, it seems likely that it concerns (mostly) monolingual L1 speakers.

## References

[B1] Abutalebi J. Green D. (2007). Bilingual language production: the neurocognition of language representation and control. *J. Neurolinguistics* 20 242–275. 10.1016/j.jneuroling.2006.10.003

[B2] Ardal S. Donald M. W. Meuter R. Muldrew S. Luce M. (1990). Brain responses to semantic incongruity in bilinguals. *Brain Lang.* 39 187–205. 10.1016/0093-934X(90)90011-5 2224493

[B3] Baayen R. Piepenbrock R. Gulikers L. (1995). *CELEX2 LDC96L14. Web Download.* Philadelphia, PA: Linguistic Data Consortium.

[B4] Bak P. Tang C. Wiesenfeld K. (1987). Self-organized criticality: an explanation of 1/f noise. *Phys. Rev. Lett.* 59 381–384. 10.1103/PhysRevLett.59.381 10035754

[B5] Balota D. Chumbley J. (1984). Are lexical decisions a good measure of lexical access? The role of word-frequency in the neglected decision stage. *J. Exp. Psychol. Hum. Percept. Perform.* 10 340–357. 10.1037/0096-1523.10.3.340 6242411

[B6] Balota D. Chumbley J. (1990). Where are the effects of frequency in visual word recognition tasks? Right where we said they were – comment. *J. Exp. Psychol. Gen.* 119 231–237. 10.1037/0096-3445.119.2.231 2141355

[B7] Berent I. Perfetti C. (1995). A rose is a reez – the 2-cycles model of phonology assembly in reading english. *Psychol. Rev.* 102 146–184. 10.1037/0033-295X.102.1.146

[B8] Carreiras M. Armstrong B. C. Perea M. Frost R. (2014). The what, when, where, and how of visual word recognition. *Trends Cogn. Sci.* 18 90–98. 10.1016/j.tics.2013.11.005 24373885

[B9] Cattell J. M. (1886). The time taken up by cerebral operations. *Mind* 11 377–392.

[B10] Coltheart M. Rastle K. Perry C. Langdon R. Ziegler J. (2001). DRC: a dual route cascaded model of visual word recognition and reading aloud. *Psychol. Rev.* 108 204–256. 10.1037/0033-295X.108.1.204 11212628

[B11] Cuetos F. Barbón A. (2006). Word naming in spanish. *Eur. J. Cogn. Psychol.* 18 415–436. 10.1080/13594320500165896

[B12] de Bot K. Lowie W. M. (2010). “On the stability of representations in the multilingual lexicon,” in *Cognitive Processing in Second Language Acquisition: Converging Evidence in Language and Communication Research* 13th Edn eds Pütz M. Sicola L. (Amsterdam: John Benjamins) 117–134.

[B13] de Groot A. Borgwaldt S. Bos M. van den Eijnden E. (2002). Lexical decision and word naming in bilinguals: language effects and task effects. *J. Mem. Lang.* 47 91–124. 10.1006/jmla.2001.2840

[B14] de Groot A. M. B. (2011). *Language and Cognition in Bilinguals and Multilinguals: An Introduction.* New York: Psychology Press.

[B15] Elman J. L. (2004). An alternative view of the mental lexicon. *Trends Cogn. Sci.* 8 301–306. 10.1016/j.tics.2004.05.003 15242689

[B16] Elman J. L. (2009). On the meaning of words and dinosaur bones: lexical knowledge without a lexicon. *Cogn. Sci.* 33 547–582. 10.1111/j.1551-6709.2009.01023.x 19662108 PMC2721468

[B17] Elman J. L. (2011). Lexical knowledge without a lexicon? *Ment. Lex*. 6 1–33. 10.1075/ml.6.1.01elm 22069438 PMC3209550

[B18] Fodor J. A. (1983). *The Modularity of Mind: An Essay on Faculty Psychology.* Cambridge, MA: MIT Press.

[B19] Frost R. (1998). Toward a strong phonological theory of visual word recognition: true issues and false trails. *Psychol. Bull.* 123 71–99. 10.1037/0033-2909.123.1.71 9461854

[B20] Gabbay D. M. (2010). *Philosophy of Complex Systems.* Oxford: Elsevier.

[B21] Gerritsen M. Meurs W. F. V. Planken B. C. Korzilius H. P. L. M. (2016). A reconsideration of the status of english in Netherlands within the kachruvian three circles model. *World Englishes* 35 457–474. 10.1111/weng.12206

[B22] Gilden D. (1997). Fluctuations in the time required for elementary decisions. *Psychol. Sci.* 8 296–301. 10.1111/j.1467-9280.1997.tb00441.x

[B23] Gilden D. (2001). Cognitive emissions of 1/f noise. *Psychol. Rev.* 108 33–56. 10.1037/0033-295X.108.1.33 11212631

[B24] Grainger J. Jacobs A. (1996). Orthographic processing in visual word recognition: a multiple read-out model. *Psychol. Rev.* 103 518–565. 10.1037/0033-295X.103.3.518 8759046

[B25] Green D. W. Abutalebi J. (2013). Language control in bilinguals: the adaptive control hypothesis. *J. Cogn. Psychol.* 25 515–530. 10.1080/20445911.2013.796377 25077013 PMC4095950

[B26] Hahne A. (2001). What’s different in second-language processing? Evidence from event-related brain potentials. *J. Psycholinguist. Res.* 30 251–266. 10.1023/A:1010490917575 11523274

[B27] Hahne A. Friederici A. D. (2001). Processing a second language: late learners’ comprehension mechanisms as revealed by event-related brain potentials. *Biling. Lang. Cogn.* 4 123–141. 10.1017/S1366728901000232

[B28] Harm M. W. Seidenberg M. S. (2004). Computing the meanings of words in reading: cooperative division of labor between visual and phonological processes. *Psychol. Rev.* 111 662–720. 10.1037/0033-295X.111.3.662 15250780

[B29] Holden J. (2005). “Gauging the fractal dimension of response times from cognitive tasks,” in *Contemporary Nonlinear Methods for Behavioral Scientists: A Webbook Tutorial* eds Riley M. A. Van Orden G. C. (Arlington, VA: National Science Foundation) 267–318.

[B30] Katz L. Feldman L. B. (1983). Relation between pronunciation and recognition of printed words in deep and shallow orthographies. *J. Exp. Psychol. Learn. Mem. Cogn.* 9 157–166. 10.1037/0278-7393.9.1.157 6220113

[B31] Kello C. T. Anderson G. G. Holden J. G. Van Orden G. C. (2008). The pervasiveness of 1/f scaling in speech reflects the metastable basis of cognition. *Cogn. Sci.* 32 1217–1231. 10.1080/03640210801944898 21585450

[B32] Kello C. T. Beltz B. C. Holden J. G. Van Orden G. C. (2007). The emergent coordination of cognitive function. *J. Exp. Psychol. Gen.* 136 551–568. 10.1037/0096-3445.136.4.551 17999570

[B33] Kroll J. Stewart E. (1994). Category interference in translation and picture naming: evidence for asymmetric connections between bilingual memory representations. *J. Mem. Lang.* 33 149–174. 10.1006/jmla.1994.1008 19084237

[B34] Kroll J. F. Bobb S. C. Misra M. Guo T. (2008). Language selection in bilingual speech: evidence for inhibitory processes. *Acta Psychol.* 128 416–430. 10.1016/j.actpsy.2008.02.001 18358449 PMC2585366

[B35] La Heij W. Hooglander A. Kerling R. van der Velden E. (1996). Nonverbal context effects in forward and backward word translation: evidence for concept mediation. *J. Mem. Lang.* 35 648–665. 10.1006/jmla.1996.0034

[B36] Lemhöfer K. Dijkstra T. Schriefers H. Baayen R. H. Grainger J. Zwitserlood P. (2008). Native language influences on word recognition in a second language: a megastudy. *J. Exp. Psychol. Learn. Mem. Cogn.* 34 12–31. 10.1037/0278-7393.34.1.12 18194052

[B37] Lowie W. Plat R. de Bot K. (2014). Pink noise in language production: a nonlinear approach to the multilingual lexicon. *Ecol. Psychol.* 26 216–228. 10.1080/10407413.2014.929479

[B38] Lupker S. (1984). Semantic priming without association – a 2nd look. *J. Verbal Learning Verbal Behav.* 23 709–733. 10.1016/S0022-5371(84)90434-1

[B39] Meschyan G. Hernandez A. E. (2006). Impact of language proficiency and orthographic transparency on bilingual word reading: an fMRI investigation. *Neuroimage* 29 1135–1140. 10.1016/j.neuroimage.2005.08.055 16242351

[B40] Perry C. Ziegler J. C. Zorzi M. (2007). Nested incremental modeling in the development of computational theories: the CDP+ model of reading aloud. *Psychol. Rev.* 114 273–315. 10.1037/0033-295X.114.2.273 17500628

[B41] Potter M. So K. Voneckardt B. Feldman L. (1984). Lexical and conceptual representation in beginning and proficient bilinguals. *J. Verbal Learning Verbal Behav.* 23 23–38. 10.1016/S0022-5371(84)90489-4

[B42] Psychology Software Tools Inc. (2006). *E-Prime 1.2.* Available at: http://www.pstnet.com

[B43] Ries S. Legou T. Burle B. Alario F.-X. Malfait N. (2015). Corrigendum to why does picture naming take longer than word naming? the contribution of articulatory processes. *Psychon. Bull. Rev.* 22 309–311. 10.3758/s13423-014-0668-4 24980218

[B44] Rodriguez-Fornells A. (2002). Electrophysiological estimates of the time course of semantic and phonological encoding during listening and naming. *Neuropsychologia* 40 778–787. 10.1016/S0028-3932(01)00188-9 11900728

[B45] Rodriguez-Fornells A. Rotte M. Heinze H. Nosselt T. Munte T. F. (2002). Brain potential and functional MRI evidence for how to handle two languages with one brain. *Nature* 415 1026–1029. 10.1038/4151026a 11875570

[B46] Segalowitz N. Poulsen C. Segalowitz S. (1999). RT coefficient of variation is differentially sensitive to executive control involvement in an attention switching task. *Brain Cogn.* 40 255–258.

[B47] Shibahara N. Zorzi M. Hill M. P. Wydell T. Butterworth B. (2003). Semantic effects in word naming: evidence from english and japanese kanji. *Q. J. Exp. Psychol. A* 56 263–286. 10.1080/02724980244000369 12613564

[B48] Smith M. Magee L. (1980). Tracing the time course of picture-word processing. *J. Exp. Psychol. Gen.* 109 373–392. 10.1037/0096-3445.109.4.373 6449530

[B49] Spivey M. (2007). *The Continuity of Mind.* Oxford: Oxford University Press.

[B50] Spivey M. Dale R. (2004). On the continuity of mind: toward a dynamical account of cognition. *Psychol. Learn. Motiv.* 45 87–142. 10.1016/S0079-7421(03)45003-2

[B51] van den Bosch A. Content A. Daelemans W. de Gelder B. (1994). Measuring the complexity of writing systems. *J. Quant. Linguist.* 1 178–188. 10.1080/09296179408590015

[B52] Van Orden G. Holden J. Turvey M. (2003). Self-organization of cognitive performance. *J. Exp. Psychol. Gen.* 132 331–350. 10.1037/0096-3445.132.3.331 13678372

[B53] Van Orden G. C. Kloos H. Wallot S. (2009). “Living in the pink: intentionality, wellbeing, and complexity,” in *Philosophy of Complex Systems: Handbook of the Philosophy of Science* Vol. 10 ed. Hooker C. (Amsterdam: Elsevier) 629–674.

[B54] Van Wijnendaele I. Brysbaert M. (2002). Visual word recognition in bilinguals: phonological priming from the second to the first language. *J. Exp. Psychol. Hum. Percept. Perform.* 28 616–627. 10.1037/0096-1523.28.3.616 12075892

[B55] Wartenburger I. Heekeren H. R. Abutalebi J. Cappa S. F. Villringer A. Perani D. (2003). Early setting of grammatical processing in the bilingual brain. *Neuron* 37 159–170. 10.1016/S0896-6273(02)01150-9 12526781

[B56] Wijnants M. L. Bosman A. M. T. Hasselman F. Cox R. F. A. Van Orden G. C. (2009). 1/f scaling in movement time changes with practice in precision aiming RID C-5603-2011. *Nonlinear Dynamics Psychol. Life Sci.* 13 79–98. 19061546

[B57] Wijnants M. L. Hasselman F. Cox R. F. Bosman A. M. Van Orden G. (2012). An interaction-dominant perspective on reading fluency and dyslexia. *Ann. Dyslexia* 62 100–119. 10.1007/s11881-012-0067-3 22460607 PMC3360848

[B58] Woollams A. M. Lambon Ralph M. A. Madrid G. Patterson K. E. (2016). Do you read how I read? systematic individual differences in semantic reliance amongst normal readers. *Front. Psychol.* 7:1757. 10.3389/fpsyg.2016.01757 27920731 PMC5118465

